# Traces of strong selective pressures in the genomes of C_4_ grasses

**DOI:** 10.1093/jxb/erw390

**Published:** 2017-01-21

**Authors:** Pascal-Antoine Christin

**Affiliations:** Animal and Plant Sciences, University of Sheffield, Western Bank, Sheffield, UK

**Keywords:** Adaptation, C_4_ photosynthesis, cross-species selection scans, evolution, gene discovery, genomics, grasses, parallel evolution, positive selection, proteins

**C_4_ photosynthesis is nature’s response to CO_2_ limitations, and evolved recurrently in several groups of plants. To identify genes related to C_4_ photosynthesis, Huang *et al.* looked for evidence of past episodes of adaptive evolution in the genomes of C_4_ grasses. They identified a large number of candidate genes that evolved under divergent selection, indicating that, besides alterations to expression patterns, the history of C_4_ involved strong selection on protein-coding sequences.**

The C_4_ syndrome relies on a series of anatomical and biochemical adaptations that function together to concentrate CO_2_ in some parts of the leaf (Hatch, 1987). This effectively boosts photosynthesis, and increases growth rates in subtropical and tropical conditions (Atkinson *et al.*, 2016). The prospect of improving non-C_4_ crops, such as rice and wheat, by engineering an efficient C_4_ cycle in them is therefore very appealing, and several projects have been set up in an attempt to realize this ambitious goal. Unfortunately, while the main enzymes of the C_4_ pathway were identified long ago and have been characterized in detail, the genetic mechanisms underlying regulation of the pathway, transport of metabolites, and leaf anatomy remain poorly understood.

Engineering a complex biochemical pathway, which requires the action and coordination of numerous proteins, is virtually impossible when some of the underlying genes are yet to be identified. Evolution successfully engineered this intricate pathway, and did it a remarkably large number of times for such a complex trait (Sage *et al.*, 2011). While the details of how this happened are still to be elucidated, the traces of this accomplishment should still be present in the genomes of extant species. Any single genome consists of a ‘long list of letters’ that is difficult to decipher, and yet the comparison of multiple genomes has the power to reveal changes that happened during evolution. Obviously, the significance of these changes is another problem, but past evolutionary pressures left specific footprints on the small fraction of genomes that correspond to protein-coding genes.

Because each amino acid can be encoded by different nucleotide triplets, some nucleotide changes (substitutions) do not affect the protein. These are synonymous substitutions, while non-synonymous substitutions change the amino acid and so result in a slightly different protein. Under a purely stochastic model, the rates of fixation of these two types of substitutions should be similar and, as such, their ratio (dN/dS) should equal one (Yang, 1998). Most non-synonymous changes will, however, be detrimental and thus preferentially removed by selection, leading to an observed dN/dS much smaller than one in most cases. Exceptionally, when a change in the catalytic properties of the encoded enzyme benefits the organism, the rate of fixation of non-synonymous substitutions will increase, leading to an observed dN/dS that can exceed one, at least for some parts of a gene. Such instances of positive selection are classically associated with ‘arms races’ between hosts and pathogens, leading to sustained elevated dN/dS throughout the history of the gene (Endo *et al.*, 1996). However, episodic changes to the catalytic environment can also increase dN/dS for limited periods, corresponding to a few branches of a phylogenetic tree. Huang *et al.* looked for such traces of past episodes of adaptive evolution linked to C_4_ photosynthesis by comparing the genomes of C_4_ and non-C_4_ grasses (Box 1).

**Box 1. Adaptive evolution in C_4_ grasses**

Phylogeny of the six species included in the analysis from Huang *et al.*: maize (*Zea mays*), *Sorghum bicolor*, *Setaria viridis* (wild progenitor of *Setaria italica*), *Dichanthelium oligosanthes*, *Brachypodium distachyon* and rice (*Oryza sativa*). Red branches indicate where the C_3_ to C_4_ transitions occurred. Photos courtesy of Pu Huang, James Schnable and Elizabeth Kellogg.

**Figure:**
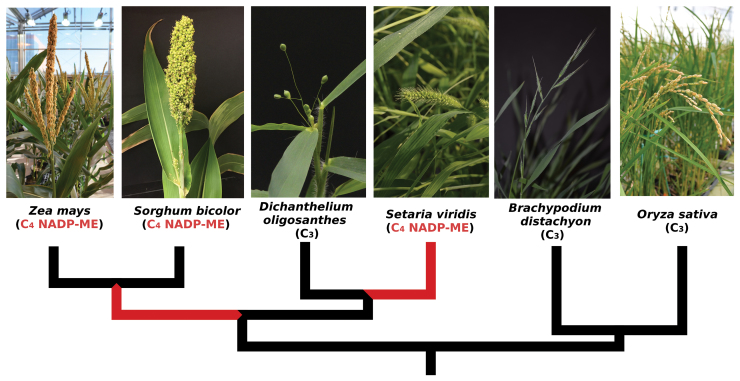

## Evidence of past positive selection reveals candidate genes for C_4_ photosynthesis

Tracking evolutionary modifications to identify changes linked to C_4_ photosynthesis is not a new idea. The many independent origins of the C_4_ trait make it particularly amenable to comparative studies, enabling identification of the ecological and physiological changes linked to its evolution (e.g. Edwards and Smith 2010; Atkinson *et al.*, 2016). In recent years, attempts to identify all of the C_4_-related genes similarly relied on evolution-based comparisons, but these mainly focused on gene expression (Brautigam *et al.*, 2014; Mallmann *et al.*, 2014). It is only more recently that attention has turned to genomic changes, such as duplication of genes (Emms *et al.*, 2016). While adaptive evolution of C_4_ enzymes involving kinetic changes has been reported (Svensson *et al.*, 2003; Christin *et al.*, 2007), one might hypothesize that this would concern only a handful of enzymes – specifically, those linked to core C_4_ reactions and their very high catalytic rates. Huang and colleagues decided to challenge this assumption and adopt bioinformatic approaches to identify all the genes that evolved under elevated dN/dS, specifically in C_4_ grasses.

After screening the genomes of six grasses, including three C_4_ taxa belonging to two independent C_4_ origins (Box 1), Huang *et al.* identified 88 genes that evolved under elevated dN/dS on branches belonging to one or several of the C_4_ lineages. This type of genome scan is inherently subject to false positives. In addition, the methodology cannot strictly differentiate between adaptive evolution and relaxed selection. Finally, the genes might have been under divergent selection along these branches for reasons other than C_4_ evolution. Fortunately, the putative link with the C_4_ trait was confirmed for a number of candidates by independent evidence, including *a priori* knowledge for a few of them and high expression in C_4_ tissues for many others. The list produced by Huang *et al.* therefore includes many promising candidates, some of which might be linked to C_4_ anatomy. If confirmed, their identification would represent a major breakthrough for the engineering of C_4_ photosynthesis into non-C_4_ crops. In the short term the results already affect the way we should envision C_4_ evolution.

## Physiological innovation through adaptive evolution of numerous protein-coding genes

For the most part, previous studies have linked phenotypic variation to alterations in gene expression and regulation (King and Wilson, 1975; Brawand *et al.*, 2011). While these have certainly also played a key role in the evolution of C_4_ photosynthesis, the new results show that the modification of promoter sequences and regulatory networks is only one part of the story, which also includes adaptive changes in the coding sequences of a large number of genes. This is impressive, providing even more evidence that the recurrent transition to C_4_ photosynthesis represents a considerable evolutionary feat. The observations of Huang *et al.* also reveal a new set of questions; in particular, why did the coding sequences of so many proteins need to be adapted, both in terms of biochemical properties and evolutionary drivers? The biochemical component of this question will remain unanswered until extensive characterization is performed, and yet hints about the evolutionary pressures can already be proposed.

The precise timing of positive selection episodes is beyond the scope of this comparative work because of the limited number of species sampled, which corresponds to the few grasses for which a complete genome is currently available. Indeed, these episodes are inferred along phylogenetic branches that expand from the last divergence of C_4_ and non-C_4_ taxa to the first divergence of two C_4_ taxa within the same lineage (Box 1). With only six species, this interval is initiated long before the transition to C_4_ photosynthesis and continues for a long period after C_4_ evolved, spanning up to 20 million years of changes. As more genomes become available, similar analyses will be able to pin down the timing of these episodes of positive selection with more precision. Until then, we can only speculate.

As with any complex trait, the numerous changes that define extant C_4_ plants were probably spread over long periods of evolutionary time, from the occurrence of capacitating mutations in non-C_4_ ancestors to changes directly responsible for the emergence of a C_4_ physiology, and continuous adaptive alterations after its origin (Christin and Osborne, 2014). Modelling efforts predict that changes in expression patterns can lead to the emergence of a C_4_ cycle in plants with C_4_-like anatomical traits (Heckmann *et al.*, 2013; Mallmann *et al.*, 2014). However, evolution did not stop after the initial transition to C_4_ photosynthesis, and the presence of a working C_4_ cycle, even if rudimentary, probably created a selective impetus for the fixation of substitutions that improved the C_4_ syndrome. The genes detected by Huang *et al.* probably underwent adaptive mutations that improved the fit of the proteins to the new catalytic environment. Their impressive number suggests that the selective pressure for improving the C_4_ syndrome was very strong or maintained over a long evolutionary period, possibly throughout the diversification of C_4_ plants.

Until now, research on C_4_ evolution has focused mainly on the events that led to a C_4_ cycle, largely ignoring those that followed its emergence. The discovery of widespread C_4_-related selection on coding genes should motivate new research into the changes that contributed to the improvement or diversification of the C_4_ syndrome. A first step in this direction is to acknowledge the diversity of C_4_-related traits within each C_4_ lineage, and design comparative experiments that capture this diversity. With continuous advances in sequencing technology, this goal might soon become achievable for comparative genomics, contributing towards a full elucidation of the changes that were selected, both before and after the first C_4_ plants emerged.
